# *DISEMM*: a tool for the investigation of elasto-plastic behaviour on polycrystalline samples using X-ray and neutron diffraction

**DOI:** 10.1107/S1600576722003314

**Published:** 2022-05-08

**Authors:** Alexander Heldmann, Michael Hofmann, Markus Hoelzel

**Affiliations:** a Heinz Maier-Leibnitz Zentrum (MLZ), Technische Universität München, Lichtenbergstraße 1, 85748 Garching, Germany

**Keywords:** single-crystal elastic constants, elasto-plastic self-consistent (EPSC) modelling, *DISEMM*, mechanical modelling

## Abstract

The software *DISEMM* is designed to analyse diffraction data from *in situ* loading experiments on polycrystalline samples for the determination of single-crystal elastic constants (SECs) and elasto-plastic self-consistent (EPSC) modelling of lattice strains. The SECs can be obtained from powder-diffraction elastic constants using a variety of grain-to-grain interaction models, namely Voigt, Reuss, Hill, Kröner, de Wit and Matthies approaches. The texture of the polycrystalline sample can be taken into account using the orientation distribution function of the grains. For the analysis of two-phase materials, an approach was implemented to calculate the stress transfer between the phases and its impact on the apparent elastic properties. The calculated SECs can then be used as input into the EPSC model, which allows the user to predict the elasto-plastic behaviour for comparison with experimental lattice strain data and to investigate the activation of individual slip systems. For this purpose, critical resolved shear stresses and hardening parameters are adapted iteratively.

## Abstract 2

*DISEMM* is a software tool to analyse neutron or X-ray diffraction data on polycrystalline samples collected under mechanical load, combining the determination of single-crystal elastic constants and the methods of elasto-plastic self-consistent modelling in one package.

## 1. Introduction

Powder-diffraction methods using laboratory X-ray diffraction, synchrotron facilities or neutron sources offer a variety of possibilities for the investigation of engineering materials. In particular, well established methods exist to analyse fractions of the constituent phases, textures, residual stresses, microstrains or particle sizes. It has been demonstrated by Hauk & Kockelmann (1979 ▸) that single-crystalline elastic constants can also be derived by diffraction studies on polycrystalline samples under external uniaxial stress using approximations for the grain-to-grain interactions. Gnäupel-Herold *et al.* (1998 ▸) took the idea further. They derived the single-crystal elastic constants of different metals of cubic structure and proposed the χ^2^ function for the minimization, which is also used in the program *Diffraction assisted mechanical modelling* (*DISEMM*) described here. The method is a kind of reverse of classical stress analysis. In particular, it requires the determination of lattice strains ε(*hkl*) of oriented grains in the elastic region as a function of the applied stress σ^L^. This technique offers the possibility to derive single-crystal elastic constants in technical alloys or multi-phase materials which are not available as single crystals. Thus, a variety of applications can be found in the literature. Detailed descriptions of the method are given elsewhere (Gnäupel-Herold *et al.*, 1998 ▸; Howard & Kisi, 1999 ▸; Matthies *et al.*, 2001 ▸; Heldmann *et al.*, 2019 ▸).

Measuring lattice strains by X-ray or neutron diffraction under mechanical load above the yield stress allows analysis of the plastic anisotropy of the material. The interpretation of the experimental data can be supported by plasticity models such as elasto-plastic self-consistent (EPSC) simulations, based on the framework proposed by Hutchinson (1970 ▸). In contrast to finite-element methods, the EPSC modelling framework relies on averaging of grain orientations to solve different equations from Eshelby’s inclusion model analytically (Eshelby, 1957 ▸; Hill, 1965*a* ▸,*b* ▸, 1966 ▸, 1967 ▸). To our best knowledge, the first applications of the elasto-plastic self-consistent approach for the analysis of diffraction studies were carried out by Tomé and co-workers (Lebensohn & Tomé, 1993 ▸; Turner & Tomé, 1994 ▸; Turner *et al.*, 1995 ▸). Since then, EPSC modelling has been widely applied for the interpretation of the evolution of lattice strains. Detailed descriptions of the EPSC approach can be found in the work of Hutchinson (1970 ▸) and in studies where the EPSC formalism is used to analyse diffraction data (Turner & Tomé, 1994 ▸; Lorentzen *et al.*, 2002 ▸; Gloaguen *et al.*, 2008 ▸; Saleh *et al.*, 2013 ▸).

*DISEMM* is a software tool to analyse neutron or X-ray diffraction data on polycrystalline samples collected under mechanical load, combining the determination of single-crystal elastic constants and the methods of elasto-plastic self-consistent modelling in one package. In the following, the features of *DISEMM* and examples of its applications are presented.

## 2. General functionality

**Figure 1:**
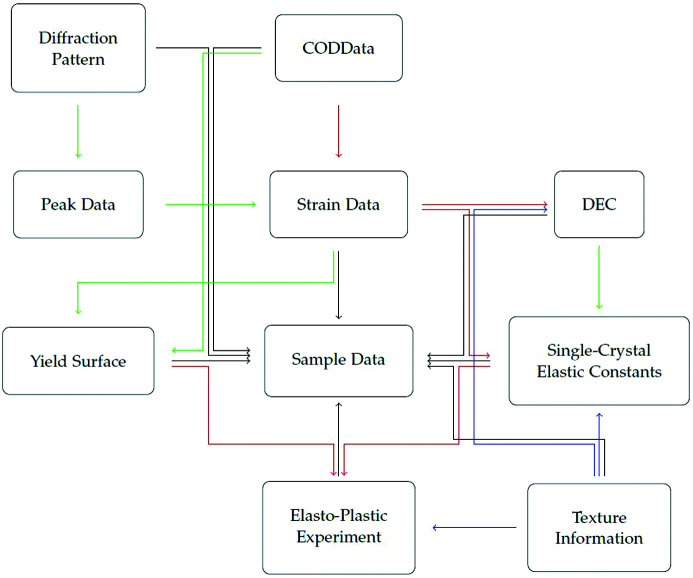
Flow chart of the program developed for the strain-data treatment. Black lines indicate the storage, for example Dif fraction Pattern is stored at Sample Data. Blue lines indicate that the inclusion of these data in the evaluation is optional. Red lines show the requirements to derive specific parameters. For example, to derive the single-crystal elastic constants the Strain Data are required. Green lines indicate an alternative way for calculation. For example, the single-crystal elastic constants may be calculated from the strains directly, or alternatively they can be determined using the polycrystalline DECs.

The basic concept of *DISEMM* is to derive all available elasto-plastic properties from experimental powder-diffraction data in as few steps as possible. In particular, diffraction elastic constants (DECs) and single-crystal elastic constants (SECs) are obtained. These properties are then used as input parameters for the elasto-plastic self-consistent model to predict the stress–strain behaviour of the investigated material. A flowchart of the main program architecture, *i.e.* classes in the sense of object-oriented programming, is given in Fig. 1 ▸. *DISEMM* contains a set of tools for the analysis of anisotropy, load transfer, texture and activated slip systems along different crystal orientations. The program is designed to harmonize strain data obtained by diffraction experiments, tensile tests and EPSC modelling. This design allows the comparison of each aspect of EPSC modelling with experimental parameters.

### 2.1. Data storage and main classes

The core of the program consists of the class Sample Data, which stores all relevant information of the sample.

Crystallographic data like cell parameters, the composition of the phase and its symmetry group as well as additional parameters regarding the phase fraction and microstructure (grain shape and size) are stored for multiphase analysis in the class CODData.

To account for macroscopic deformation, multiple tensile tests may be added for the evaluation and optionally matched with the diffraction experiment for the comparison to predicted stress–strain curves.

Texture data may be loaded in the form of an orientation distribution function into the Sample Data class and used instead of the isotropic approach when the grains are randomly oriented.

The parameters of the yield surface are stored in the resources file in XML format for the implemented crystal symmetries and may be edited before launch. For each individual slip system the yield strength and hardening parameters can be edited manually. Optionally, a custom set of possible active slip systems may be used.

### 2.2. Strain data

**Figure 2:**
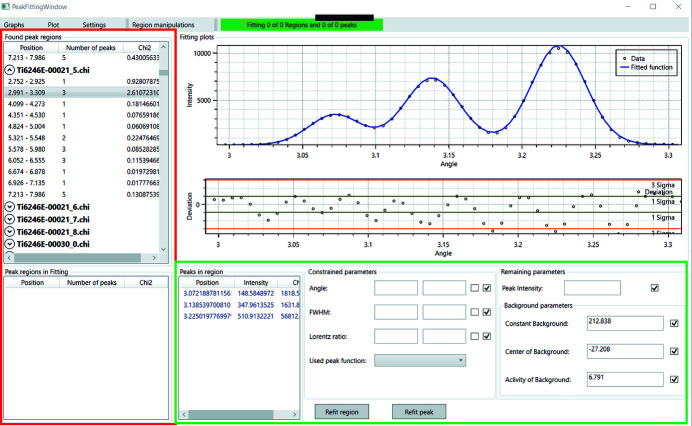
Screenshot of the peak-fitting window of *DISEMM* during the evaluation. On the left in the red box there is a list of peaks contained in regions. The selected region is shown in the plot, where the blue line indicates the fitted curve. In addition, the height and width of each peak can be adjusted manually in the green box. Each fit is performed in its own thread. Therefore, a large number of regions can be fitted simultaneously. When a diffraction pattern is added to the Sample Data, each pattern is searched for peaks. These are combined into regions and automatically fitted. Starting values are improved each time a peak is fitted.

The class Strain Data is one of the basic classes needed to derive the elastic properties of the investigated sample. It consists of a measured strain ε(*hkl*), which is associated with a Bragg peak *hkl*, and its orientation with respect to the experimental setup. The data can be either added directly via an ASCII file or retrieved straightforwardly by fitting 1D diffraction patterns. The peaks are automatically scanned by a detection routine and fitted while the pattern data are added to Dif fraction Pattern. *DISEMM* offers the possibility to improve the automated fits by manually adjusting and refitting a peak (see Fig. 2 ▸).

The Bragg reflections are described by three different peak functions, Gaussian, Lorentzian and pseudo-Voigt, where the last is set as standard with 90% Gaussian fraction. The fit of the pattern data is performed by a Levenberg–Marquard fitting algorithm (LMA) (Press *et al.*, 2011 ▸). Starting values such as peak position and peak height are estimated from the pattern and Caglioti peak-width parameters *U*, *V* and *W* from the corresponding instrument resolution function (Caglioti *et al.*, 1958 ▸). For large data sets, the starting values may be set from previous fits to accelerate the automated fitting. In the case of overlap, the peaks are grouped and simultaneously fitted as a linear combination.

The strain values are calculated relative to the reference position of the lowest applied stress, *i.e.* 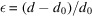 . This way, nonlinearities caused by internal stresses are minimized since existing phase- and micro-stresses, which do not change during elastic deformation, are taken into account (Behnken, 2003 ▸; Heldmann *et al.*, 2019 ▸).

### 2.3. Single-crystal elastic constants

**Figure 3:**
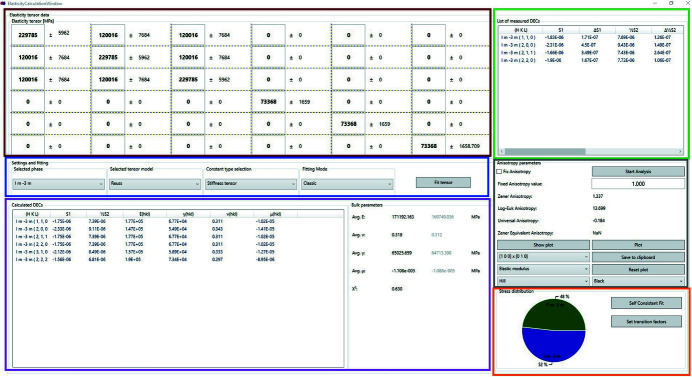
Screenshot of the evaluation of the single-crystal elastic constants. At the top left in the dark-red box the derived elastic constants are listed. Below in the blue box the settings for the analysis, such as the displayed phase, the grain-to-grain interaction model, and whether stiffnesses or compliances should be displayed, are selected. Below in the purple box the DECs predicted by the selected model are shown. On the right, average values of the Young, shear and bulk moduli are given according to the used grain-to-grain interaction model; the grey text colour indicates the values obtained by the measurement. In the green box each experimentally obtained DEC is listed. In the grey box the analysis of the anisotropy is performed. The plot settings for parameters such as Young’s modulus and the shear modulus along different crystal directions are found here, too. In the right corner inside the orange box the transition factors for the phases are displayed if a load transfer analysis is applied.

The window managing the SEC and dedicated elastic parameters is shown in Fig. 3 ▸. As indicated in Fig. 1 ▸, *DISEMM* allows the use of two different routes to derive the single-crystal elastic constants from the experimental data. The first minimizes the differences between the strains directly as shown in equation (3), upper row, and the other minimizes the differences between the diffraction elastic constants as in equation (3), lower rows. *DISEMM* supports a total of five different grain-to-grain interaction models, suggested by Reuss, Hill, Matthies, Kroener and de Wit, for the evaluation (Voigt, 1928 ▸; Reuss, 1929 ▸; Hill, 1952 ▸; Matthies *et al.*, 2001 ▸; Kroener, 1958 ▸; de Wit, 1997 ▸).

Basically, as for classical stress analysis, the relation between the measured strains in a diffraction experiment, the diffraction elastic constants *s*_1_ and 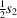 , and the stress tensor components is given by

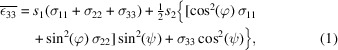

where ε_33_ is the strain measured along the scattering vector during a diffraction experiment and σ_*ii*_ are the stress tensor components applied to the sample. The orientation parameters (φ, ψ) used in *DISEMM* follow the definition provided by Heldmann *et al.* (2019 ▸).

In the following we consider a diffraction experiment under uniaxial load with σ_33_ ≠ 0 and σ_11_ = σ_22_ = 0. To derive the diffraction elastic constants, *DISEMM* sorts the experimental data to match equation (2) derived from (1):

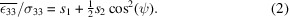

**Figure 4:**
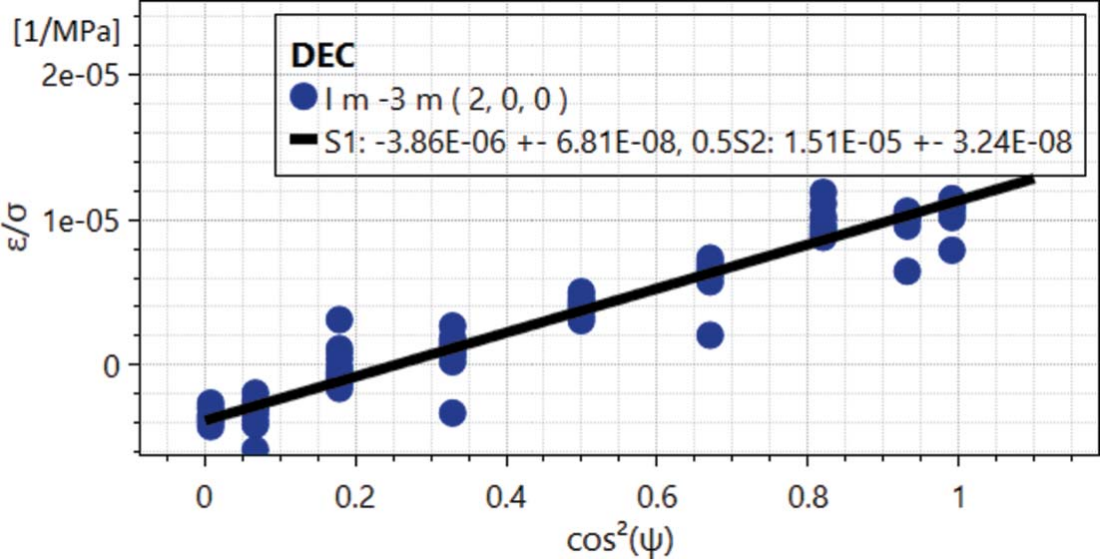
Example fit of the DEC of the 200 Bragg reflection of the β phase in the dual-phase alloy Ti–6Al–2Sn–4Zr–6Mo.

The *y* values of the data points are given by the measured strain divided by the applied stress, and the *x* values are calculated from the orientation ψ (Heldmann *et al.*, 2019 ▸). An example of the data and the fit are given in Fig. 4 ▸. The χ^2^ functions used during the LMA routine to fit the single-crystal elastic constants *a*_*ijkl*_ are given in equation (3):

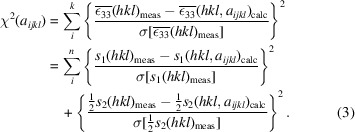

To account for texture, additional weightings are introduced during the χ^2^ minimization routine.

The software allows the user to fix the anisotropy during the fit of the SEC and to plot parameters such as Young’s or shear modulus along different crystallographic directions afterwards. It also implements different measures for the anisotropy besides the Zener anisotropy factor (Zener, 1936 ▸; Chung & Buessem, 1967 ▸; Ranganathan & Ostoja-Starzewski, 2008 ▸).

### 2.4. Dual-phase approach

In materials containing more than one phase, the grain-to-grain interaction models discussed earlier do not cover the interactions between the different phases. If the phases have significantly different rigidity, the stress appearing in the sample distributes differently among those phases. As a result, only the effective stiffness of the corresponding phase is observed during diffraction experiments. *DISEMM* implements a self-consistent scheme to calculate the stress distribution between two or more phases. First, the effective elastic constants of all constituent phases are derived directly from the measured lattice strains. With these constants, the overall average phase stress 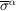 of phase α is calculated from the applied stress σ^L^ in equation (4): 

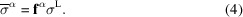

The transition factors **f** are derived from the Eshelby inclusion model according to equation (5), and therefore one phase has to be declared by the user as the inclusion I and one as the matrix M (Eshelby, 1957 ▸; Behnken, 2003 ▸).

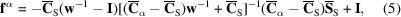

where 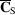 , 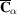 and 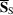 are the sample and phase averages of the elastic stiffnesses and compliances. **w** is the Eshelby tensor defined in equation (6) for a sphere in a homogeneous matrix (Eshelby, 1957 ▸):

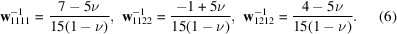

Equation (5) shows that the stress distribution in the elastic region depends on the mean single-crystal elastic constants of both phases, 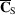 , and the mean value of phase α, 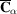 . The transition factors of the phases weighted by their phase fraction *p* ^α^ must add up to unity, *i.e.* they follow 

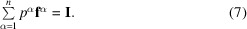

The stress–strain data are adjusted according to the transition factors. After each loop the difference between the overall average phase stresses in successive loops decreases, and the calculation loop is stopped after the changes are smaller than a certain value and convergence is reached (Heldmann *et al.*, 2019 ▸).

### 2.5. Elasto-plastic modelling

**Figure 5:**
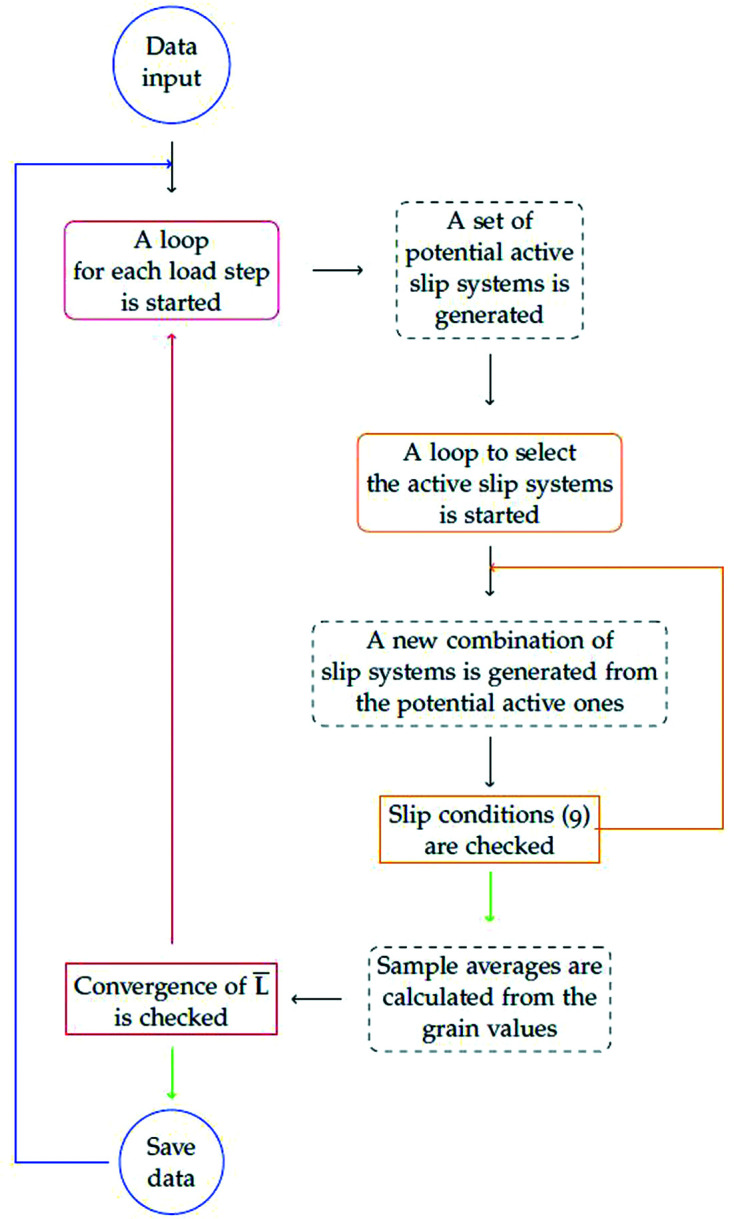
The EPSC modelling scheme requires a list of second-order stress or strain tensors as input parameters. They represent corresponding stress or strain states of the sample. The scheme consists of two loops. The first iterates until 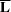 converges self-consistently and does not change from iteration to iteration. The inner, second loop ensures that only the correct combination of slip systems is active.

The elasto-plastic self-consistent modelling scheme implemented in *DISEMM* and shown in Fig. 5 ▸ is based on Hill’s work solving Eshelby’s inclusion problem (Eshelby, 1957 ▸; Hill, 1965*a* ▸,*b* ▸, 1966 ▸, 1967 ▸; Hutchinson, 1970 ▸). In its basic formalism, it requires only a few material-specific input parameters beyond those required to derive the single-crystal elastic constants. The critical resolved shear stress 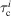 and hardening for each slip family is needed to calculate the activated slip systems of the differently oriented single crystals in the polycrystalline sample. Each increment of the stress–strain curve is then calculated by averaging over all available orientations. The stress–strain state of each orientation is saved individually, allowing the input and analysis of any given texture and its changes during plastic deformation.

The flowchart shown in Fig. 5 ▸ depicts the scheme implemented in *DISEMM* to calculate the stress–strain behaviour. The simulation input is split into individual calculation steps, and for each stress or strain state an individual loop is started. On the basis of the previous deformation history, a new set of potentially active slip systems is created for each predefined input state. The list of potential slip systems is chosen from all available slip systems of the given crystal symmetry according to which slip system satisfies

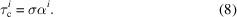

From this set the active systems meeting requirement (9) is determined:

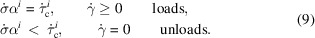

If the resolved stress rate 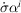 on the *i*th slip system equals the yield and the change in the shear rate is positive, the slip system loads, *i.e.* activates; otherwise it unloads. The set of active slip systems is used to calculate new iterations of the instantaneous stiffness coefficients until the difference between the iterations is smaller than a user-given value. This large calculation loop is depicted in Fig. 5 ▸. The stress–strain data of the simulation are finally stored to the Elasto-Plastic Experiment class, and a new loop for the next input step is initiated, as indicated by the blue line. A detailed introduction to the EPSC model implemented in *DISEMM* is given by various authors (Hutchinson, 1970 ▸; Turner & Tomé, 1994 ▸).

### 2.6. Data display and export

*DISEMM* supports a variety of display and export functions. Either file outputs are given in the .xlsx formatting of Microsoft *Excel* or the data may be exported in a .txt file in ASCI-II encoding. The plotting areas support direct export to the picture formats .jpeg and .png.

## 3. Results

The dual-phase approach to derive single-crystal elastic constants is shown by the example of Ti–6Al–2Sn–4Zr–6Mo, which consists of a hexagonal close-packed (h.c.p.) α phase and a body-centred cubic (b.c.c.) β phase (Heldmann *et al.*, 2019 ▸). An example of elasto-plastic self-consistent modelling is given for ferritic steel S235JR in this work.

### 3.1. Single-crystal elastic constants

Results obtained with *DISEMM* for the ferrous metals showed good agreement with existing literature data for the grain-to-grain interaction models used for the evaluation (Heldmann *et al.*, 2019 ▸). During the same analysis, it has been found that the texture in those samples does not influence the single-crystal elastic constants above the uncertainties, and therefore the isotropic approach is adequate for most cases (Heldmann *et al.*, 2019 ▸).

In the same work, the dual-phase approach was extended to more complex alloy systems like Ti–6Al–2Sn–4Zr–6Mo with great success. Here, it was possible to determine for the first time all eight single-crystal elastic constants of the α and β phases of a dual-phase titanium alloy in a single experiment. A significant load transfer from the β phase to the stiffer α phase was observed. Applying the load-transfer correction, the elastic constants of the β phase were significantly shifted to lower values and showed an excellent agreement with corresponding data obtained on the pure β alloy Ti–3Al–8V–6Cr–4Zr–4Mo. In addition, the load-transfer-corrected elastic constants of the α phase in Ti–6Al–2Sn–4Zr–6Mo matched well with the results determined for the α phase in Ti–6Al–4V (Heldmann *et al.*, 2019 ▸).

### 3.2. Elasto-plastic self-consistent modelling

**Figure 6:**
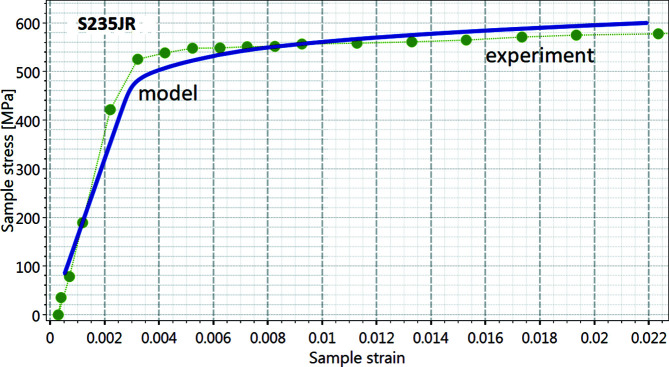
Comparison between measured macroscopic stress–strain values (green dots) and the EPSC simulation (blue line) for the steel S235JR. As expected, the yield stress increases faster for the measurement than the prediction.

The EPSC prediction of the stress–strain behaviour of the ferritic steel S235JR using *DISEMM* was compared with experimental data. The materials-science diffractometer STRESS-SPEC (Brokmeier *et al.*, 2011 ▸) was used for these diffraction studies. Fig. 6 ▸ shows the macroscopic stress–strain relation calculated by the EPSC model compared with experimental data obtained during the diffraction experiment. The single-crystal elastic constants *c*_11_ = 240 (6) GPa, *c*_12_ = 218 (6) GPa and *c*_44_ = 106 (3) GPa were derived with *DISEMM* and used as input for the modelling, resulting in a slight mismatch in Young’s modulus of about 7%. The main discrepancies are found at the onset of the plastic regime between 400 and 550 MPa. Here the simulated values show higher strain values than those from the diffraction data. In later stages of deformation, the largest mismatch of the strain rate is found at around 1% strain, but it rapidly adjusts to the correct strain rate, resulting in a slightly higher yield strength of approximately 20 MPa in this case.

**Figure 7:**
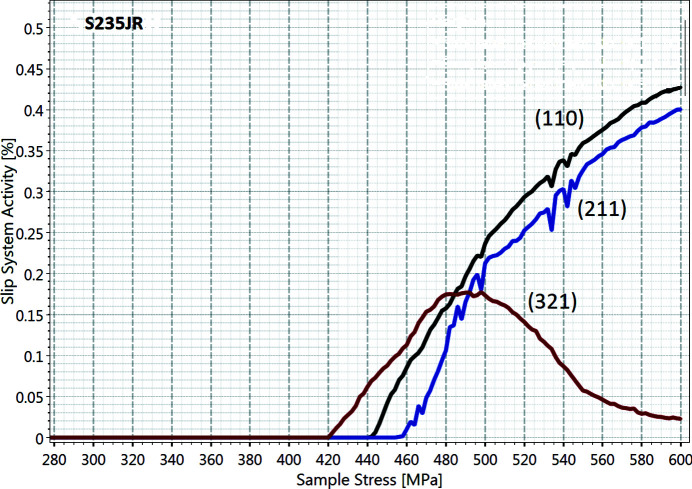
Plot of slip system activity versus macroscopic stress for the steel S235JR. The (321) slip family (red line) is activated first, followed by the (110) slip family (black line), before finally family (211) (blue line) is activated. The macroscopic strains are mainly caused by the slips on (110) and (211).

The steel S235JR exhibits a b.c.c. crystal structure and therefore the plastic strains are introduced by the activitation of the three slip families listed in Table 1 ▸. The critical resolved shear stresses of the slip families optimized during our modelling process to achieve the observed yield strength are also listed in Table 1 ▸. The higher yield stress of the (321) slip plane is due its smaller Schmid factor. The slip activity of each family is shown in Fig. 7 ▸ and is defined in this context as the ratio of active slip systems compared with all possible active systems. The (321) slip plane triggers the plastic deformation, causing only small changes in the plastic strain rate, followed by the (110) slip plane approximately 50 MPa later. The last slip family activated is (211), which starts to take over (321) which decreases its activity after reaching 500 MPa. This triggers the late deformation stage with a high increase of the plastic strain rate.

**Figure 8:**
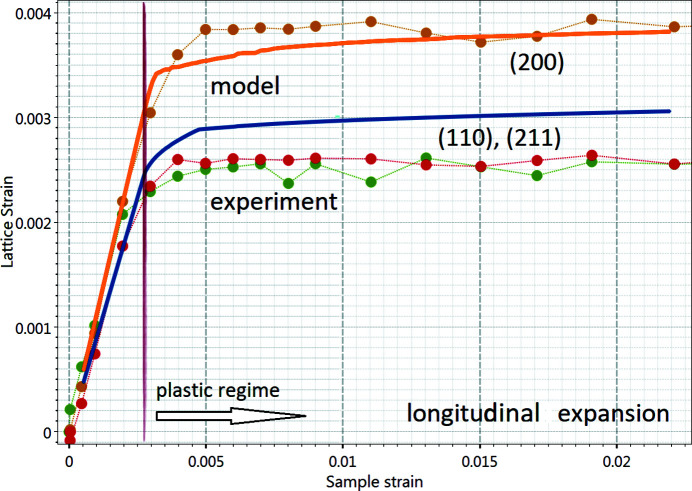
Comparison of lattice strains measured in S235JR along the longitudinal expansion (strains along load direction), marked by dots, with predictions by the EPSC model (lines). Red: (110) planes; green: (211) planes; orange: (200) planes. The lattice strain uncertainties purely derived from peak fitting are smaller than the dot size.

The lattice strains of the (110), (200) and (211) planes are shown in Fig. 8 ▸, where they are compared with values obtained from EPSC modelling. The experimental data show the highest lattice strains along (200). The EPSC model predicts the lattice strains of (200) within the uncertainties, showing some deviations when entering the plastic area. The experimental data indicate a rather gradual transition into the plastic regime, while the model predicts a more defined entry. This discrepancy from experimental data is partly caused by the cyclic loading during the experiment.

Plastic deformation occurs when the applied stress surpasses a critical amount. If the acting stress remains constant during this period of time, the deformation is driven by small changes in the stress states of the grains interacting with each other. This means that during non-continuous loading experiments additional strains will be introduced into the sample. For the current example, about 5 min times for the recording of diffraction patterns at each loading step have to be considered. In consequence, during each loading step, additional macroscopic plastic strains are introduced into the sample after reaching the plastic region during the experiment. Therefore, the measured macroscopic strains will appear larger for each loading step during the experiment. A future option will address this issue by taking into account the stress pile-up on grain boundaries for cyclic loading as suggested by Lorentzen *et al.* (2002 ▸).

**Figure 9:**
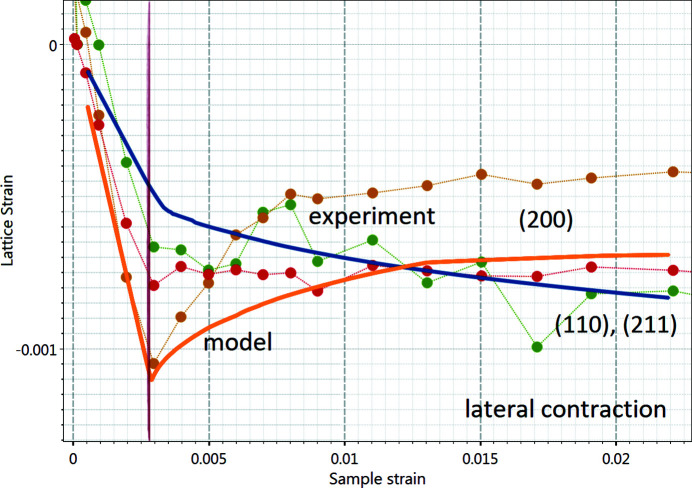
Comparison of lattice strains measured in S235JR along the lateral contraction (direction perpendicular to the load axis), marked by dots, with predictions by the EPSC model (lines). Same colour coding as in Fig. 8 ▸, but different scale for the lattice strains. The model predicts the trends well: for example, the reduction of stress on the (200) plane after entering the plastic regime.

The lateral contractions for the same (110), (200) and (211) planes are shown in Fig. 9 ▸. The anisotropy differs to some extent from the experimental data along the lateral direction because the strains for the (200) plane are predicted to be higher while the strains of (110) and (211) planes are in good agreement with the experimental data. However, the trend of the (200) plane is predicted correctly until entering the plastic regime, and only in later stages of deformation are the lattice strains measured during the experiment underestimated by about one-third.

Nevertheless, the plastic regimes show essentially the same behaviour as a similar ferritic steel reported by Daymond & Priesmeyer (2002 ▸), *i.e.* after reaching the critical yield stress the strains evolve faster than observed during the experiment. At higher strain values, the simulation catches up with the experimental values again (Daymond & Priesmeyer, 2002 ▸). The same applies to measured lattice strains during diffraction experiments where the (200) plane shows the lowest value of Young’s modulus and highest yield during tensile testing. Even the lateral contraction shows a similar decrease of the measured strains of (200), although the decrease in the α-iron phase is more distinct (Daymond & Priesmeyer, 2002 ▸).

## 4. Conclusion

*DISEMM* implements a wide range of tools to evaluate diffraction data from *in situ* loading experiments. It implements routines to fit single-crystal elastic constants from single- and multi-phase alloys considering texture and the stress distribution among different phases from diffraction data recorded *in situ* during a tensile test. It provides packages to visualize the anisotropy together with measures for quantifying the degree of anisotropy, such as for example the Zener anisotropy and the universal anisotropy index. The program also contains a package implementing a routine for elasto-plastic self-consistent modelling using the elastic evaluation results as input values. *DISEMM* is designed to harmonize experimental and simulation data and enable comparison between the two, from the macroscopic stress–strain curves to lattice strain data. Thus it offers a unique analysis tool for the investigation of polycrystalline deformation behaviour.

*DISEMM* was validated successfully on ferrous metals and applied to dual-phase titanium alloys with great success to determine all eight single-crystal elastic constants of the h.c.p. and b.c.c. phases by taking into account the stress distribution (load transfer) among the phases. An example of the EPSC model implemented is shown as a case study on the experimental data of S235JR. In summary, the EPSC framework predicts the observed deformation quite accurately and reproduces the observed stress states. In accordance with available literature, the calculated macroscopic strain shows minor discrepancies compared with the experiment at the onset of the plastic regime. However, the lattice strains of the model and experiment are in good agreement in the early plastic regime but show a more isotropic straining in the late plastic regime along the (200) direction than experimentally observed.

## 5. Availability

The software is published on the web site of the Heinz Maier-Leibnitz Zentrum (MLZ) (https://mlz-garching.de/spodi/de) and the source code is available on GitHub (https://github.com/Gipfelgrab/DISEMM/releases/). The software is also available on request from the author.

## Figures and Tables

**Table 1:** Input parameters for the different slip families of the EPSC simulation

Slip family	Yield strength (MPa)	Hardening (MPa)
(110) 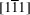	225	100
(211) 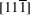	235	75
(321) 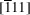	410	500
